# Impact of Total Ionizing Dose on Radio Frequency Performance of 22 nm Fully Depleted Silicon-On-Insulator nMOSFETs

**DOI:** 10.3390/mi15111292

**Published:** 2024-10-24

**Authors:** Zhanpeng Yan, Hongxia Liu, Menghao Huang, Shulong Wang, Shupeng Chen, Xilong Zhou, Junjie Huang, Chang Liu

**Affiliations:** 1 Key Laboratory for Wide Band Gap Semiconductor Materials and Devices of Education, School of Microelectronics, Xidian University, Xi’an 710071, China; 23111213430@stu.xidian.edu.cn (Z.Y.); slwang@xidian.edu.cn (S.W.); spchen@xidian.edu.cn (S.C.); xilzhou@163.com (X.Z.); huangjunjie2024@163.com (J.H.); 2 Science and Technology on Reliability Physics and Application Technology of Electronic Component Laboratory, China Electronic Product Reliability and Environmental Testing Research Institute, Guangzhou 511370, China

**Keywords:** fully depleted silicon-on-insulator (FD-SOI), RF (radio frequency), total ionizing dose (TID), *f_max_* (maximum oscillation frequency), *f_T_* (cut-off frequency)

## Abstract

In this paper, the degradation mechanism of the RF performance of 22 nm fully depleted (FD) silicon-on-insulator nMOSFETs at different total ionizing dose levels has been investigated. The RF figures of merit (the cut-off frequency *f_T_*, maximum oscillation frequency *f_max_*) show significant degradation of approximately 14.1% and 6.8%, respectively. The variation of the small-signal parameters (output conductance (*g_ds_*), transconductance (*g_m_*), reflection coefficient (|Γ_in_|), and capacitance (*C_gg_*)) at different TID levels has been discussed. TID-induced trapped charges in the gate oxide and buried oxide increase the vertical channel field, which leads to more complex degradation of small-signal parameters across a wide frequency range.

## 1. Introduction

As the size of semiconductor processes continues to shrink, the impact of various non-ideal effects on the performance of semiconductor devices is becoming increasingly severe. Against this backdrop, the significant suppression of short channel effects by the 22 nm ultra-thin bulk body and buried oxide (UTBB) fully depleted silicon-on-insulator (FDSOI) technology has attracted attention [1]. Additionally, due to its ultra-thin top silicon film and thinner buried oxide layer (BOX), FDSOI technology exhibits unique advantages in combating single particle effects [2,3], which also makes these devices highly attractive and competitive for integrated circuits in aerospace and military applications. Moreover, with the advancement of silicon-based CMOS technology, the use of silicon-based MOSFETs in the radio frequency (RF) market is expanding [4]. Compared to bulk silicon RF-CMOS devices, SOI RF-CMOS devices have better leakage current control and short channel effect suppression, providing unique advantages in RF applications [5].

However, due to the presence of the BOX layer, FDSOI devices are still affected by the total ionizing dose (TID) effect. In the fully depleted silicon-on-insulator (FD-SOI) technology, the buried oxide (BOX) is an additional insulator capable of trapping TID charges, and when energetic charged particles pass through the BOX, the deposited energy generates electron–hole pairs escaping the initially complexed holes into stationary oxide charges and causing the transistor threshold voltages to drift through the strong coupling effect between the front and back gates of the UTBB FD-SOI devices [6,7]. Compared to bulk silicon CMOS devices, the charge trapping in the oxide layer makes the total dose effect in SOI devices more complex [8]. Furthermore, previous studies have shown that SOI devices exposed to total ionizing dose (TID) radiation in space environments experience significant changes in their RF characteristics [9,10]. Therefore, for FDSOI devices applied in the aerospace and military fields, investigating the impact of the total ionizing dose (TID) effect on their radio frequency (RF) characteristics holds significant research value. Although earlier studies have reported the total dose effect of SOI RF-CMOS devices at different process sizes [11,12,13], mostly focusing on 130 nm, 90 nm, 60 nm, and 45 nm SOI devices, there is still a lack of research on the degradation of RF characteristics caused by the TID effect in 22 nm UTBB-FDSOI MOSFETs.

In this work, we focus on exploring the specific effects of total dose irradiation on the RF performance of the 22 nm UTBB-FDSOI device, relying on the technical computer-aided design (TCAD) tool. In order to deeply reveal the intrinsic mechanism of device RF performance degradation under total dose irradiation, we first performed an exhaustive three-dimensional (3D) numerical simulation of the total dose effect using the Sentaurus Synopsys framework. Subsequently, we performed a detailed AC small-signal simulation analysis on the total-dose irradiated device samples. Through this process, we successfully extracted and analyzed changes in key small-signal parameters, including output conductance (*g_ds_*), transconductance (*g_m_*), the reflection coefficient (|Γ_in_|), and capacitance (*C_gg_*) after irradiation. Variations in these parameters are directly related to, and significantly affect, the overall performance of the device in high-frequency operating scenarios.

## 2. Simulation Settings

### 2.1. Device Structure

In this paper, we utilize the SDE tool from the Sentaurus TCAD simulation platform to build a 3D N-type FDSOI device model based on the 22 nm process node, with device structure parameters sourced from the process library.

**Figure 1:**
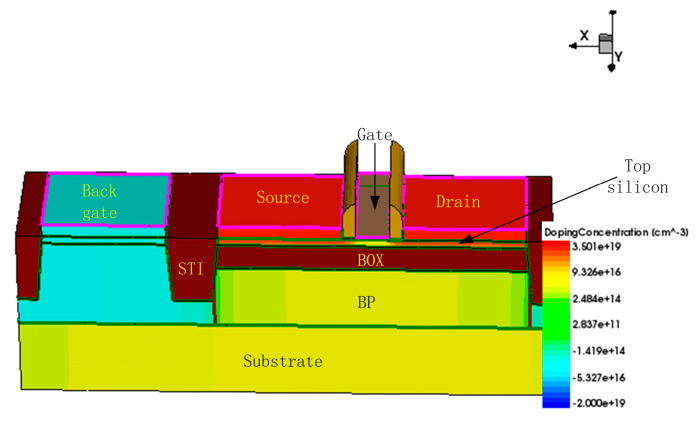
Schematic diagram of 22 nm FDSOI device.

Under the 22-nanometer Global Foundries process, the top silicon film thickness of the device is 6 nm, and the BOX layer thickness is 20 nm. Because HfO_2_ is a high-K dielectric that can effectively reduce gate leakage currents in FDSOI devices, 1 nm of HfO_2_ high-K dielectric combined with 1 nm of SiO_2_ is used as the gate oxide. The structure of the 3D FDSOI device is illustrated in Figure 1. The minimum channel length of the device is 20 nm, and the width-to-length ratio of 80 nm/22 nm is selected based on the dimension data provided by the process library. Contacts are defined for the source (left red region), drain (right red region), gate (orange region), and back gate (blue region) of the device to facilitate subsequent simulation studies. The BOX layer is located in the brown area directly beneath the source, drain, and gate, while the other brown areas are STI isolation layers. Since the FDSOI device does not have a floating body effect, body contacts are not required for this device.

The doping information of the device is closely related to its electrical characteristics; however, this information is kept strictly confidential by the process manufacturers. Therefore, the doping concentration can only be adjusted and tested based on experience and the design manual.

In this work, in order to improve the hot carrier effect of the device, the source–drain light doping technique is used. The doping concentration in the lightly doped source–drain region is 7 × 10^15^ cm^−3^, using Gaussian doping. The source–drain doping concentration is 5 × 10^19^ cm^−3^, also using Gaussian doping, while the substrate doping concentration is 5 × 10^15^ cm^−3^, and the doping method is uniform doping. Based on the dimensional data provided by the process library and adjusted by the design manual, the finalized device parameters are shown in Table 1.

**Figure 2:**
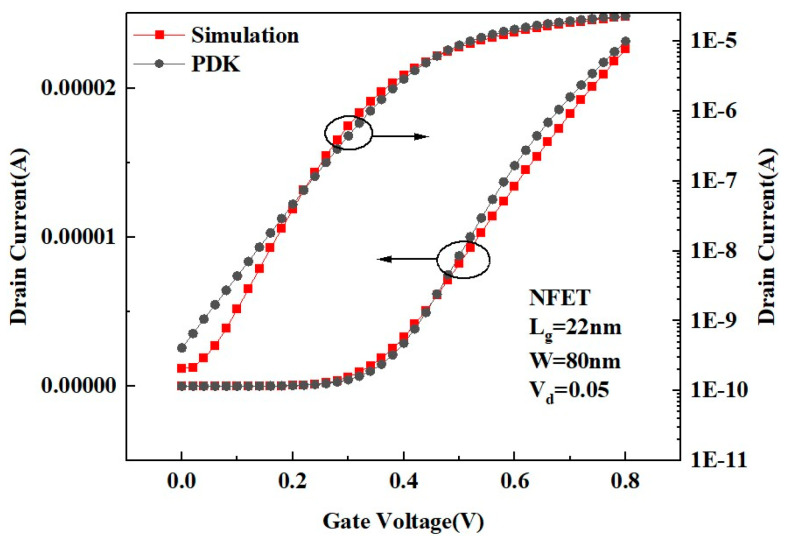
Comparison of FDSOI drain current transfer characteristics with process library experimental data (PDK).

For the established 3D FDSOI structural model, this work performs an exhaustive electrical characterization simulation analysis. During the simulation, we set the drain voltage to 0.05 V and systematically adjust the gate voltage of the NMOS to gradually increase it from 0 V to 0.8 V. To ensure the accuracy of the simulation results, we refer to the NMOS electrical characteristic curves in the SPICE (Simulation Program with Integrated Circuit Emphasis) model of the same size in Cadence software 19.1, meticulously calibrating the established FDSOI model. By comparing the actual measurement data with the simulation results, we successfully realized the accurate calibration of the device. The calibrated electrical characteristic curves are shown in Figure 2. From Figure 2, it can be clearly observed that the electrical characteristic curves of the established 3D FDSOI model closely align with the reference model (PDK) on both ordinary (left axis) and logarithmic (right axis) scales, demonstrating an excellent match. The DC electrical parameters extracted from the curves in Figure 2 are listed in Table 2.

### 2.2. Simulation Mechanism and Method

Silicon dioxide material subjected to high-energy particle bombardment will generate electron–hole pairs. Some of these electron–hole pairs will quickly recombine within a short time, while the uncombined pairs will drift under the influence of the electric field. Due to the differences in mobility, the electrons are quickly swept out of the oxide layer, whereas the holes with lower mobility will drift more slowly in the oxide layer. During this process, some holes are captured by traps in the SiO_2_, forming stable positively charged oxide trap charges. Other holes move to the interface, where they participate in reactions that generate interface state trap charges [14]. In 22 nm (UTBB-FDSOI) MOSFETs, the degradation of device characteristics due to high TID effects is mainly affected by the trapped charges in the buried oxide (BOX) layer, while the TID effects in shallow trench isolation (STI) and spacer oxides are negligible [15].

We use the Gamma Radiation Model in Sentaurus for TID effect simulation and add it to the Physics module in Sdevice. Since Sentaurus cannot directly solve the equations for insulating materials (e.g., SiO_2_), the materials of the BOX layer and gate oxide in the model are replaced with OxideAsSemiconductor to enable carrier transport properties similar to those of semiconductors, and the carrier mobility in the .par file is modified. Furthermore, the Trap model is added to the Physics module in Sdevice to simulate the generation process of trapped charges in the oxide layer of the FDSOI device. In actual radiation environments, the total ionizing dose effect primarily has significant impacts on the device’s oxide layer. Other materials, such as metals and semiconductors, are also affected, but to a lesser extent. Therefore, in the simulation process, we mainly focus on the significant performance changes exhibited by the FDSOI device due to the impact on its oxide layer under radiation exposure. In Sentaurus, electron–hole pairs generated due to irradiation are described using (1) and (2).

(1) $$G_{r}=g_{0}D\cdot Y(F)$$

(2) $$Y\left(F\right)={\left(\frac{F+E_{0}}{F+E_{1}}\right)}^{m}$$

where *D* is the dose rate, *E*_0_, *E*_1_, and m are constants, *g*_0_ is the electron–hole pair generation rate, *F* is the electric field, and *G_r_* is derived by using the dose rate as a linear function. According to the Sentaurus user manual, the values of *E*_0_, *E*_1_, *m*, and *g*_0_ are 0.1, 1.35 × 10^6^, 0.9, and 7.6 × 10^12^, respectively. The operating voltage (V_DD_) of the device is 0.8 V. The radiation source simulated by the radiation physics model is a γ-ray, with a dose rate set to 100 rad (SiO_2_)/s. During the entire irradiation process, the device bias state is OFF (the drain bias is at V_DD_ and the other sub-terminals are grounded), which has been proven to be the worst bias state of the device in previous work [16]. The AC small-signal simulation was performed before and after irradiation reached 300 krad(SiO_2_), and RF S-parameters were measured.

**Figure 3:**
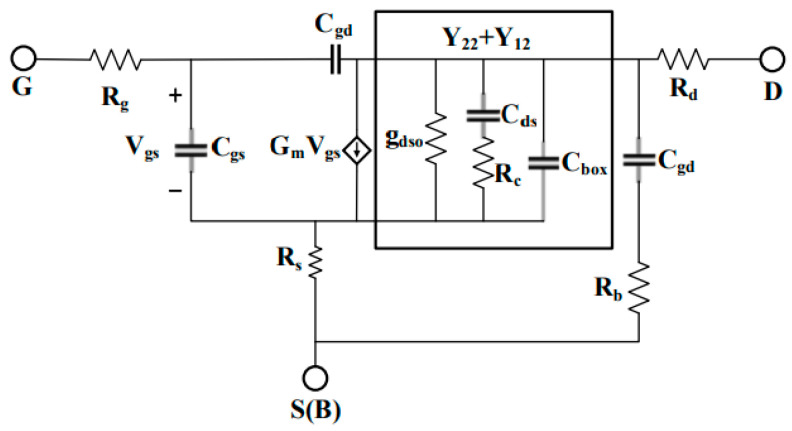
FDSOI small-signal equivalent circuit diagram.

**Figure 4:**
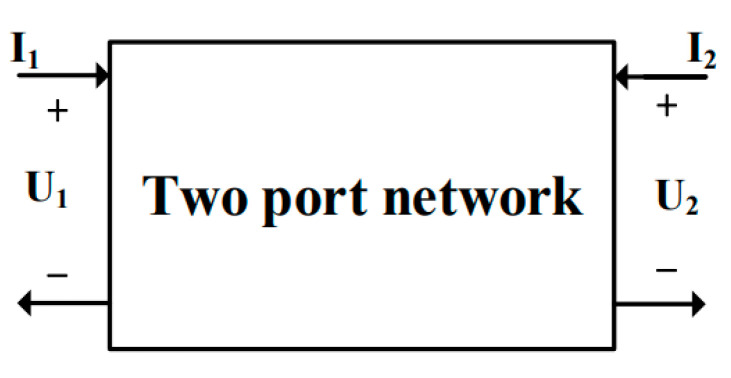
Two-port network schematic.

As for the RF AC small-signal simulation, based on the small-signal equivalent circuit diagram shown in Figure 3, the gate and drain of the device can be considered as a two-port network when its source and substrate are grounded, as shown in Figure 4. This two-port network is characterized using two independent vector currents, *I*_1_ and *I*_2_, and two independent vector voltages, *U*_1_ and *U*_2_. To investigate the RF characteristics of the UTBB-FDSOI device, a gate voltage can be selected as the static operating point and a small AC signal can be applied to the gate for simulation. Various RF parameters of the device, such as the impedance parameter (Z), admittance parameter (Y), and hybrid parameter (H), can be obtained through AC small-signal simulation. These RF parameters can effectively reflect the performance of the device under RF conditions. In this work, the RF parameters are measured in the frequency range from 0.1 GHz to 1000 GHz under the saturation condition (*V_ds_* = V_DD_) at different TID levels.

**Figure 5:**
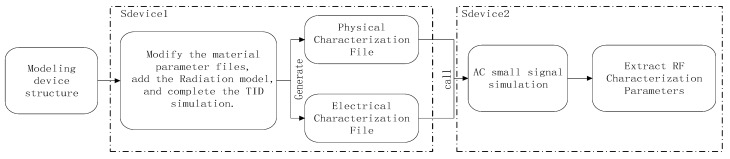
Schematic diagram of the simulation process of TID coupled with AC small signal.

The process of introducing the radiation effect on the FDSOI device and performing AC small-signal simulation is illustrated in Figure 5. The entire simulation process is based on the Sdevice tool. After building the device structure model and completing the calibration using the SDE tool, TID simulation is first carried out in Sdevice1, and the simulation results are saved to generate the corresponding files. Then, in Sdevice2, the result files from the TID simulation are called to perform the AC small-signal simulation, and finally, the RF characteristic parameters after irradiation are extracted.

## 3. Results and Discussion

### 3.1. Influence of TID Effect on the DC Characteristics

**Figure 6:**
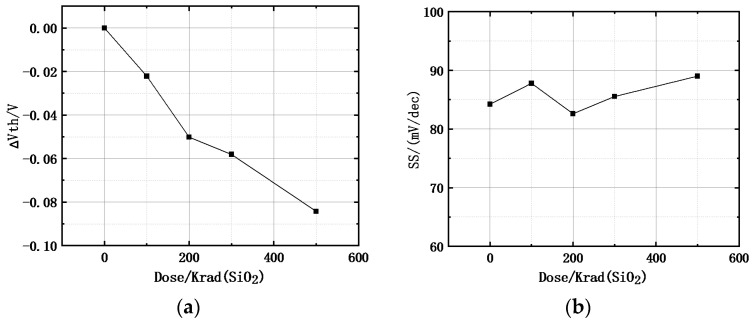
Threshold voltage drifts (**a**) and subthreshold swing drifts (**b**) vs. different doses under OFF-bias for the FDSOI nMOS.

During the simulation of the TID effect on the UTBB-FDSOI device, the threshold voltage drift and subthreshold swing drift under different total ionizing doses were extracted, as shown in Figure 6.

From Figure 6a, it can be seen that the threshold voltage of the 22 nm UTBB-FDSOI device exhibits a negative drift under irradiation, and the magnitude of the drift increases with increasing irradiation doses. From Figure 6b, it is clear that the subthreshold swing of the 22 nm UTBB-FDSOI device shows only a small change (less than 10%) under different irradiation doses, remaining within the range of 80 mV/dec to 90 mV/dec. This is because the BOX layer in UTBB-FDSOI devices is relatively thin, providing some resistance to total ionizing dose irradiation, and improves the overall stability of the device.

### 3.2. Influence of TID Effect on the RF Characteristics

The RF S-parameters are important for characterizing the RF properties of a device. Important RF characteristics of a device (characteristic frequency *f_T_* and maximum oscillation frequency *f_max_*) can be extracted from the measured S-parameters. Therefore, accurately extracting these parameters is essential.

For the H-parameter, the two-port network can be expressed in terms of the input current *I*_1_ and output current *I*_2_, based on the input current *I*_1_ and output voltage *U*_2_, which can be represented in matrix form. *h*_21_ denotes the current amplification when the output is short-circuited, i.e., current gain. The characteristic frequency *f_T_* is defined as the frequency at which the current gain *h*_21_ is 0 dB. The *f_T_* is related to the speed of the device, and the higher the characteristic frequency, the faster the response of the device [17].

(3) $$\left[\begin{array}{c} V_{1}\\ I_{2} \end{array}\right]=\left[\begin{array}{cc} h_{11} & h_{12}\\ h_{21} & h_{22} \end{array}\right]\left[\begin{array}{c} I_{1}\\ V_{2} \end{array}\right]$$

(4) $$h_{21}=\frac{I_{2}}{V_{1}}{|}_{V_{2}=0}$$

The Mason gain formula is the logarithmic value of the ratio of the output signal to the input signal, as shown in Formula (5), where *G* is the Mason unit power gain (MUG), *P_out_* is the output power, and *P_in_* is the input power. The maximum oscillation frequency, *f_max_*, is defined as the frequency at which the Mason unit power gain, MUG, is 0 dB.

(5) G=10log⁡PoutPin

**Figure 7:**
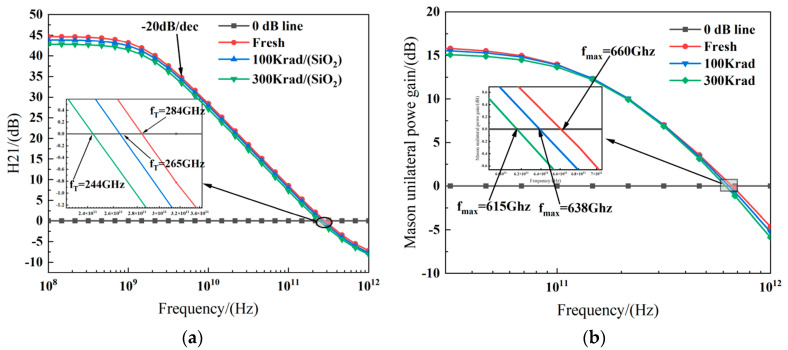
Current gain *h*_21_ (**a**) and Mason’s unilateral power gain (**b**) vs. frequency at different TID levels tested at *V_ds_* = 0.8 V and *V_gs_* = 0.8 V for the FDSOI nMOS.

Figure 7a,b illustrate the relationship between the current gain (*h*_21_) and Mason unit power gain (MUG) with frequency at different dose levels. It can be observed that after 300 krad irradiation, *f_T_* and *f_max_* decrease by approximately 14.1% and 6.8%. Notably, *f_T_* exhibits a greater degradation amplitude compared to the characteristic frequency *f_max_*.

According to the small-signal equivalent circuit diagram in Figure 3, *f_T_* and *f_max_* can be expressed as follows [18]:

(6) fT=gm2πCgs1+CgdCgs+Rs+RdCgdCgsgm+gd+gd≈gmCgs+Cgd

(7) $$f_{max}\approx \frac{f_{T}}{2\sqrt{\left(R_{s}+R_{g}\right)\cdot g_{ds}+2\pi R_{g}f_{T}C_{gd}}}$$

From Equation (6), we know that the characteristic frequency *f_T_* is inversely proportional to *C_gs_* + *C_gd_* and directly proportional to the high-frequency transconductance *g_m_*. We use the total gate capacitance *C_gg_* to represent the sum of *C_gs_* and *C_gd_* (i.e., *C_gg_* = *C_gs_* + *C_gd_*). From Equation (7), it is evident that the maximum oscillation frequency *f_max_* is related to parameters such as output conductance *g_ds_*, *f_T_*, and gate resistance. It can be seen that *f_T_* and *f_max_* depend on several frequency-related parameters (transconductance *g_m_*, output conductance *g_ds_*, gate capacitance *C_gg_*, and gate resistance *R_g_*). In this work, we extracted these frequency-dependent parameters at different TID levels. These parameters were obtained by analyzing the S-parameters measured at the DC operating point (*V_ds_* = 0.8 V and *V_gs_* = 0.8 V), while subtracting the effects of parasitic components and extracting them using the Bracale method.

*C_gg_*, *g_m_*, and *g_ds_* can be obtained from the imaginary part of *Y*_11_, the real part of *Y*_21_, and the real part of *Y*_22_, respectively. The matrix equation for the Y-parameter is shown in Equation (8).

(8) $$\left[\begin{array}{c} I_{1}\\ I_{2} \end{array}\right]=\left[\begin{array}{cc} Y_{11} & Y_{12}\\ Y_{21} & Y_{22} \end{array}\right]\left[\begin{array}{c} V_{1}\\ V_{2} \end{array}\right]$$

**Figure 8:**
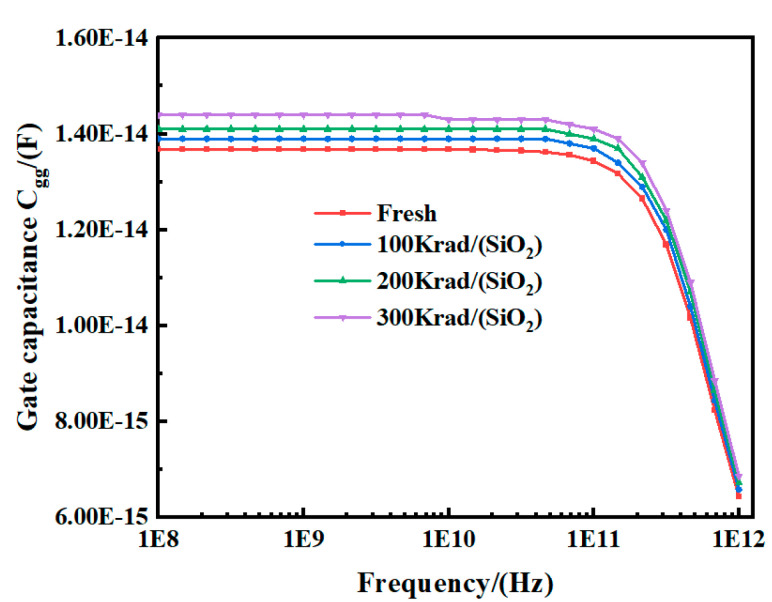
Gate capacitance extracted by imaginary part of *Y*_11_ variation with frequency at different TID levels tested at *V_ds_* = 0.8 V and *V_gs_* = 0.8 V for the FDSOI nMOS.

As shown in Figure 8, the gate capacitance *C_gg_* versus frequency for different TID levels is presented. As the TID level increases, the gate capacitance tends to increase in the frequency range below 100 GHz (by approximately 5.3%), while the effect of increasing TID levels on the gate capacitance is not significant at frequencies above 100 GHz. This can be attributed to the fact that the electron–hole pairs generated at the interface between the buried oxide and the trench are trapped by interfacial defects under the influence of the TID effect, while irradiation-introduced charges accumulate in the gate-sensitive region. The hole accumulation region at the interface between the trench and the buried oxide layer exerts an electric field influence on the device trench, which leads to an increase in the gate–drain capacitance. In addition, the decay trend of the gate capacitance is similar to the transconductance *g_m_*, which may be related to variations in the gate resistance.

**Figure 9:**
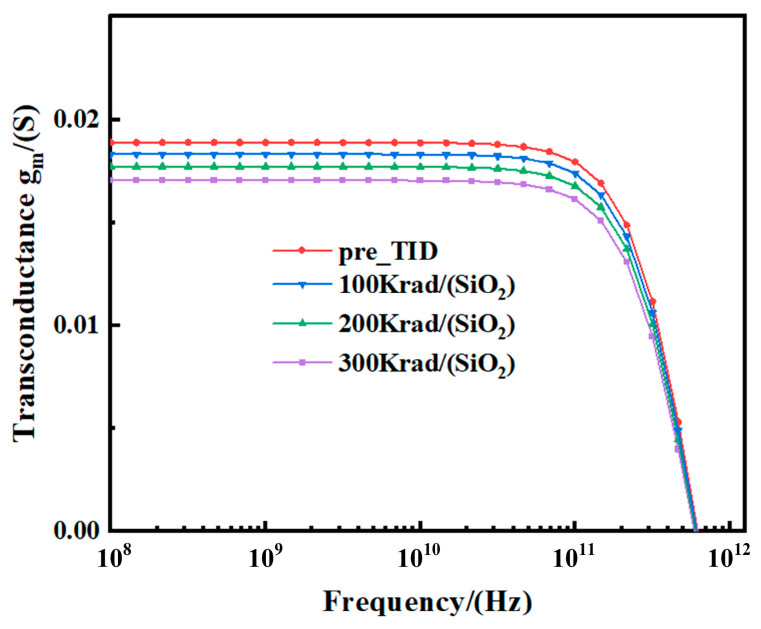
Extracted *g_m_* (real (*Y*_21_)) with frequency at different TID levels tested at *V_ds_* = 0.8 V and *V_gs_* = 0.8 V for the FDSOI nMOS.

Figure 9 shows the variation of *g_m_* (i.e., the real part of *Y*_21_) with frequency for different radiation doses. It can be observed that with the increase in the TID level, *g_m_* shows a decreasing trend (approximately 9.7%) in the frequency range below 100 GHz, while the variation of *g_m_* is not significant in the frequency range above 100 GHz. It is noteworthy that the variation trend of *g_m_* is similar to that of the gate capacitance. In addition, as demonstrated in Equation (6), the decrease in *g_m_* and *C_gg_* due to the increased TID level is responsible for the reduction in the characteristic frequency *f_T_*. The *f_T_* is critical in RF characterization because once the device operates beyond this frequency, the RF parameters of the device begin to degrade. As shown in Figure 8 and Figure 9, both pre- and post-irradiation *g_m_* and *C_gg_* begin to degrade rapidly as they approach the characteristic frequency.

**Figure 10:**
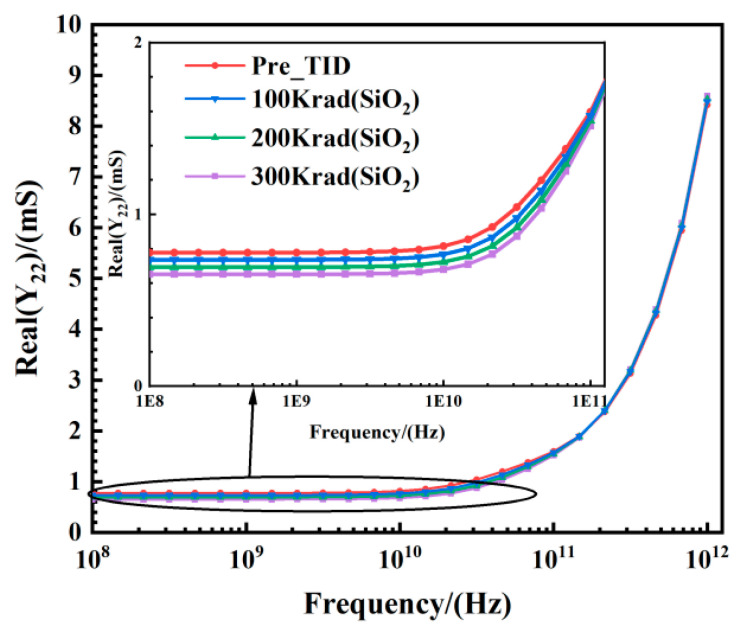
Output conductance extracted by real (*Y*_22_) vs. frequency at different TID levels tested at *V_ds_* = 0.8 V and *V_gs_* = 0.8 V for the FDSOI nMOS.

An important factor contributing to the decrease in the maximum oscillation frequency is the change in the output conductance *g_ds_*. The output conductance *g_ds_* can be extracted from the real part of *Y*_22_, as shown in Figure 10. It can be seen that in the frequency range from 1 × 10^8^ Hz to 1 × 10^10^ Hz, the *g_ds_* starts to decrease as the TID level increases. However, at lower frequencies, the output conductance decreases with increasing TID levels, while in the higher frequency range (from 1 × 10^11^ Hz to 1 × 10^12^ Hz), the increase in the TID level causes *g_ds_* to rise, albeit slightly. It is well known that the floating body effect is absent in FDSOI devices, but this advantage is not apparent when the device is operated at high frequencies. The decrease in output conductance under high-frequency conditions can be attributed to the presence of body resistance.

**Figure 11:**
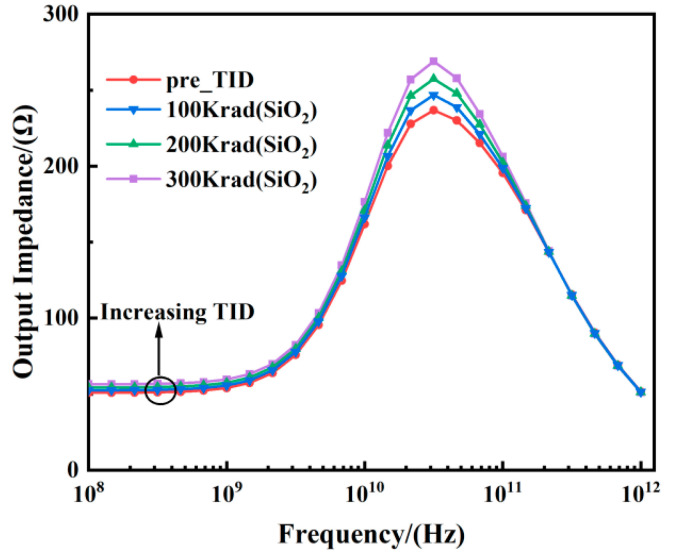
Output impedance extracted by real (*Z*_22_) vs. frequency at different TID levels tested at *V_ds_* = 0.8 V and *V_gs_* = 0.8 V for the FDSOI nMOS.

Figure 11 shows the variation of output impedance *Z*_22_ with frequency for different TID values. At low frequencies, the output impedance increases with rising TID values (approximately 11%). In the frequency range between 1 × 10^10^ Hz and 1 × 10^11^ Hz, the output impedance reaches a peak, and the increase in TID levels leads to a more pronounced degradation of *Z*_22_. At frequencies greater than 1 × 10^11^ Hz, the degradation of *Z*_22_ with increasing TID levels is minimal and negligible. The increase in output impedance implies a decrease in output conductance, which can be demonstrated by the decreasing output conductance with increasing TID levels.

**Figure 12:**
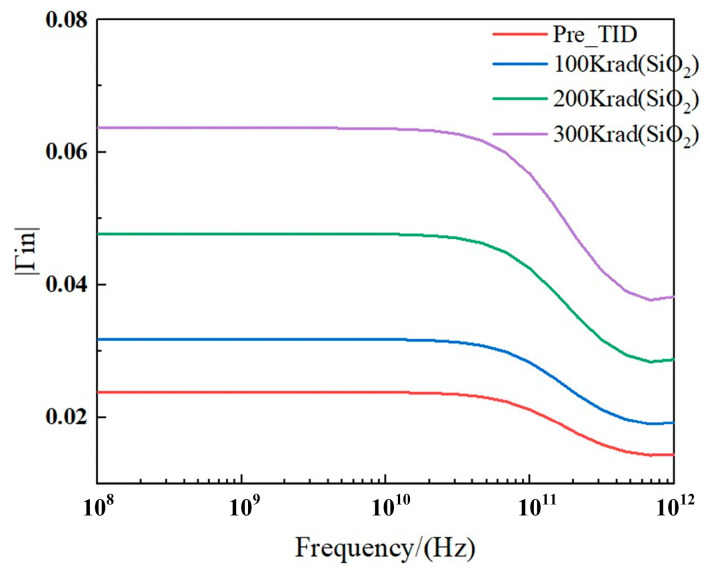
Reflection coefficient vs. frequency at different TID levels tested at *V_ds_* = 0.8 V and *V_gs_* = 0.8 V for the FD SOI nMOS.

In RF applications, the DC operating point drift caused by TID effects can lead to the degradation of RF performance and mismatches between RF components. Such mismatches can, in turn, result in an increase in the terminal reflection coefficient (Γ_in_). The increase in the terminal reflection coefficient due to TID effects is illustrated in Figure 12. The simulation results indicate that the parameter drift caused by TID can lead to mismatches between otherwise matched RF modules, thereby increasing the reflection coefficient between these modules. For example, for FDSOI NMOS devices, when the TID dose reaches 300 krad(SiO_2_) at a frequency of 100 GHz, |Γ_in_| increases by 0.041.

**Figure 13:**
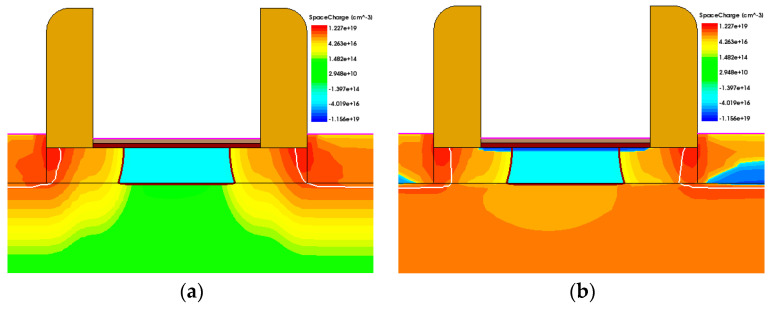
Distribution map of radiation-induced charges in the buried oxide layer and channels (**a**) before and (**b**) after 300 krad irradiation.

By taking a cross-section perpendicular to the *Y*-axis at the junction between the side gate and drain region, a two-dimensional cross-section of the FDSOI device can be obtained. Figure 13 shows the distribution of radiation-induced charges in the buried oxide layer before and after irradiation. The darker the color, the more electron–hole pairs are generated in this region during irradiation. The electron–hole pairs generated at the interface between the buried oxide layer and the channel are captured by interface defects, which in turn affect the channel. Therefore, we pay special attention to the distribution of induced charges at the interface between the channel and the buried oxide layer.

From Figure 13, it can be observed that irradiation generates a large number of electrons and holes in the buried oxide layer. Among these, holes are captured by defects at the upper and lower interfaces of the buried oxide layer, resulting in regions of hole accumulation. The hole accumulation region at the interface between the channel and the buried oxide layer applies an electric field to the device’s channel. A strong vertical electric field at the buried oxide layer interface causes the effective carriers to scatter in the channel, leading to a decrease in mobility and a degradation of *g_m_*.

**Figure 14:**
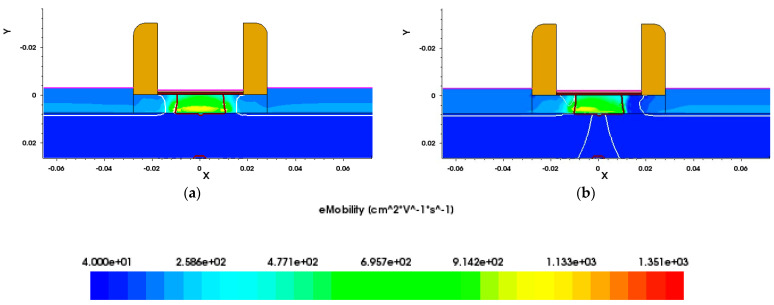
Electron mobility distribution in channel (**a**) before and (**b**) after irradiation with 300 krad.

Figure 14 shows the electron mobility distribution in the channel at different irradiation doses. As can be seen in Figure 14, the range of high electron mobility in the channel after irradiation is significantly reduced compared with that before irradiation, especially at the Si/SiO_2_ interface, where the electron mobility decreases most significantly, which suggests that as the irradiation dose increases, the probability of carriers colliding with the Si/SiO_2_ interface also increases, leading to a corresponding decrease in electron mobility. Furthermore, we can see from Figure 14 that the electron mobility near the drain region decreases more significantly than that near the source region after irradiation. This is because, during the irradiation process, the device is biased at *V_ds_* = 0.8 V and *V_gs_* = 0.8 V, resulting in a stronger electric field and higher current density near the drain region. This exacerbates electron collisions and scattering, increases heat accumulation, and raises the occurrence of defects introduced by irradiation in that area. Consequently, this leads to an asymmetric change in the channel after irradiation, where the electron mobility in the region near the drain decreases more significantly than in the region near the source.

In order to analyze the changes in the electron mobility of the devices during irradiation in greater detail, a two-dimensional plane at *Y* = 0 (i.e., along the direction of the channel) was intercepted for comparative analysis. This plane is able to visualize the changes in the channel region of the device during irradiation.

**Figure 15:**
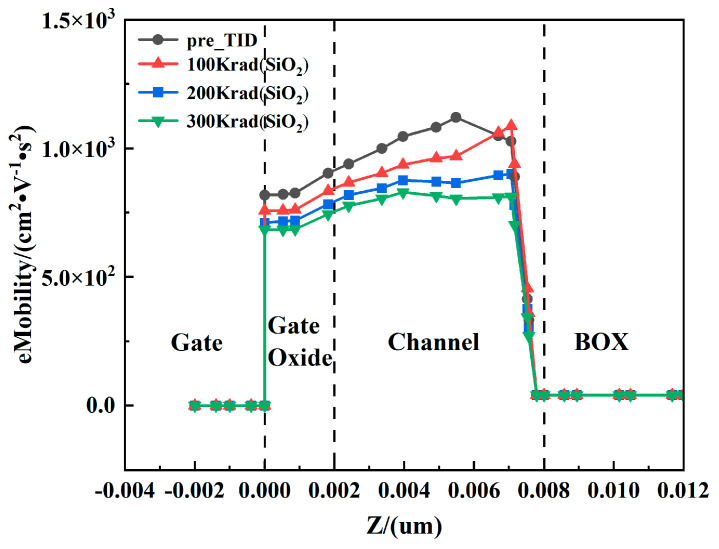
Variation of electron mobility along the *Z*-axis at X = 0.

The variation of electron mobility in the *Z* direction is analyzed in detail, as shown in Figure 15. Along the *Z*-axis, the region from 0.000 µm to 0.002 µm corresponds to the gate oxide layer, while the region from 0.002 µm to 0.008 µm corresponds to the channel area, with a channel thickness of 0.006 µm. It can be observed that the electron mobility exhibits a clear degradation trend with increasing total dose levels. In particular, the degradation of electron mobility is especially pronounced in the *Z*-direction region close to the BOX layer. This is mainly due to the enhanced scattering effects experienced by the electrons at the interface between the channel and the buried oxygen layer. The results indicate that the electron mobility in the channel continues to decrease with increasing dose levels.

## 4. Conclusions

In this work, we investigate the effect of different total ionizing dose (TID) levels on the RF characteristics of the 22 nm UTBB-FDSOI device. Our results indicate that as the TID level increases, both the current gain and Mason power gain degrade. Furthermore, by extracting the characteristic frequency *f_T_* and maximum oscillation frequency *f_max_*, it was found that both the *f_T_* and *f_max_* of the FDSOI device show significant degradation due to total dose irradiation. We further analyze the variations in the gate capacitance, high-frequency transconductance, output conductance, output impedance, and reflection coefficient of the device. The results demonstrate that high-frequency transconductance, output conductance, and the reflection coefficient decrease with increasing TID levels, while gate capacitance and output impedance gradually increase.

It can be concluded that the degradation of the RF characteristics of the device is attributed to TID-induced trap charges in the gate oxide and BOX layer. The strong vertical electric field at the interface of the BOX layer leads to the scattering of the effective carriers in the channel, resulting in a decrease in carrier mobility and high-frequency transconductance *g_m_*. This leads to the degradation of the RF characteristics of the device.

## Figures and Tables

**Table 1:** Device parameters used to simulate FDSOI NFET devices.

Parameter	NFET
Gate length (L_g_, nm)	22
Gate width (W_NW_, nm)	80
Channel thickness (T_si_, nm)	6
Gate oxide thickness (T_ox_, nm)	1
Buried oxide thickness (T_box_, nm)	20
Source/Drain length (L_s_/L_d_, nm)	38
Channel doping (N_ch_, cm^−3^)	1 × 10^15^ (acceptor) 5 × 10^19^ (donor)
Source/Drain doping (N_S_/N_D_, cm^−3^)	
Gate work function ($\varphi_{m}$, eV)	4.52

**Table 2:** DC parameters of analog devices.

Parameter	NFET
Sub-threshold slope (SS, mV/dec)	84.37
Maximum transconductance (*g_m_*, *_max_*, μS)	285.20
On-state current (I_on_, μA)	91.85
Off-state current (I_off_, pA)	443.10

## Data Availability

The original contributions presented in the study are included in the article, further inquiries can be directed to the corresponding author.
